# Body mass Index does not impact long-term survival of patients with idiopathic pulmonary fibrosis undergoing lung transplantation

**DOI:** 10.3389/frtra.2023.1146779

**Published:** 2023-08-23

**Authors:** Entela B. Lushaj, Malcolm M. DeCamp, James Maloney, Glen Leverson, Nilto De Oliveira, Daniel McCarthy

**Affiliations:** ^1^ Department of Surgery, University of Wisconsin School of Medicine and Public Health, Madison, WI, United States; ^2^ Department of Surgery, Medical College of Wisconsin, Milwaukee, WI, United States

**Keywords:** lung transplant, outcomes, body mass index, pulmonary fibrosis, survival

## Abstract

**Objective:**

We investigated the impact of body mass index (BMI) on post-operative outcomes and survival of patients with interstitial pulmonary fibrosis (IPF) undergoing lung transplantation.

**Methods:**

We retrospectively reviewed 222 patients with IPF that underwent lung transplant (LT) at our institution from 2005 to 2019. Recipients were divided in 4 groups: group-1 consisted of underweight patients (BMI ≤18.5 kg/m^2^), group-2 of normal weight patients (BMI 18.5–25 kg/m^2^), group-3 of over-weight patients (BMI 25–29.9 kg/m^2^) and group-4 of obese patients (BMI ≥30 kg/m^2^).

**Results:**

Group-1 consisted of 13 (6%) patients, group-2 of 67 (30%) patients, group-3 of 79 (36%) patients, group-4 consisted of 63 (28%) patients. Median BMI for group-1 was 17 [interquartile range (IQR): 17, 18], for group-2 was 23 (22, 24), for group-3 was 29 (28, 29.5) and group-4 was 32 (31, 33). Patients in group-1 were significantly younger (*p* < 0.01). Single LT comprised the majority of operation type in group-2 to group-4 and it was significantly higher than group 1 (*p* < 0.01). Median follow-up time was 39 months (13–76). A total of 79 (35.5%) patients died by the end of study. Overall, five deaths occurred in group-1, 17 in group-2, 33 in group-3, and 24 in group-4. Kaplan–Meier analysis showed that mortality was not statistically significant between the groups (*p* = 0.24). Cox-regression analysis was used to assess other possible risk factors that could influence the effect of BMI on mortality, including transplant type (single, double), lung allocation score, and age, diabetes and creatinine levels at surgery. None of these factors were shown to affect patient mortality (*p* > 0.05). Overall reasons for death included graft failure (24%), infection (23%), respiratory failure (14%), and malignancy (13%).

**Conclusions:**

Body mass index does not impact long-term survival of patients with IPF undergoing lung transplantation.

## Introduction

Idiopathic pulmonary fibrosis (IPF) is the most common adult form of interstitial lung disease (ILD), characterized by a progressive deterioration of lung function leading to death (1). The disease progress could be slow and gradual over many years or accelerate to a rapid decline of respiratory function. Although, anti-fibrotic therapy has been shown to slow the lung function decline over time, IPF continues to be a progressive disease with poor prognosis, with a median survival time from diagnosis of 3–5 years (2–6). Although many obstacles to lung transplant (LT) remain, such as the shortage of donor lungs, opportunistic infections, and allograft rejection, LT is the ultimate treatment for patients with progressive loss of pulmonary function due to progressive pulmonary fibrosis. Due to the relative shortage of organs, the growing number of patients on the waiting list, and the increasing number of deaths during the wait for organs, the lung allocation score (LAS) was implemented in 2005 by the Organ Procurement and Transplantation Network (OPTN 7, 8). Studies have shown that LAS appears to be achieving its objectives by reducing waitlist time and altering the distribution of lung disease being transplanted on the basis of medical necessity (9). According to the data from the International Society for Heart and Lung Transplantation (ISHLT), IPF represents 40% of lung transplants. Of those 72% were bilateral lung transplantations and 28% were single lung transplants (10). Lung transplantation in itself is associated with disease-specific challenges and ISHLT has set a list of major and relative contraindications for listing patients for lung transplantation (11).

A few studies have found a correlation of BMI with patient outcomes and survival after LT and have indicated that preoperative BMI optimization is reflected in better outcomes (12–18). The purpose of our study was to investigate if pre-transplant BMI has any effect on post-operative outcomes and survival of patients in a selective group of patients with interstitial pulmonary fibrosis (IPF) undergoing lung transplantation.

## Patients and methods

A total of 386 patients received lung transplants (LT) between November, 2007 and August, 2019 at the University of Wisconsin Hospital and Clinics. Of those, 222 (57.5%) patients had interstitial pulmonary fibrosis (IPF) as their indication for LT and were the subject population for our study.

This study was approved by our Institutional Review Board (IRB). A waiver of the need to obtain consent from patients was approved. All transplant were performed in strict compliance with the ISHLT ethics. Patient baseline characteristics and peri-operative data were prospectively acquired and maintained in our IRB approved database. Follow-up survival data were retrospectively collected from each patient's electronic medical record.

Patients were divided in 4 groups based on their body mass index (BMI): group 1 consisted of underweight patients with BMI ≤18.5 kg/m^2^, group 2 of normal weight patients with BMI 18.5–25 kg/m^2^, group 3 of overweight patients (BMI 25–29.9 kg/m^2^) and, group 4 of obese patients (BMI ≥30 kg/m^2^). Primary outcome was long-term patient survival. Secondary outcomes were post-operative complications including prolonged ventilation (ventilation lasting >24 h post-surgery), pneumonia, primary graft dysfunction, air leaks and unplanned hospital readmissions. Overall survival time was calculated from the date of transplant to the date of last follow-up visit or death.

### Statistical analysis

Continuous variables are represented as mean ± standard deviation, and categorical variables are represented as number and percentage. Continuous variables were compared using one-way ANOVA. Categorical variables were compared using Kruskal–Wallis test to determine group differences. If the test showed there were differences between the 4 groups, the Mann–Whitney test was used for pairwise comparisons. Overall survival time was calculated from the date of transplantation to the date of last follow-up visit or death. Cumulative event rates were calculated by the method of Kaplan and Meier. *p*-values less than 0.05 (two-sided) were considered statistically significant. All analyses were performed using the IBM SPSS statistical software program (IBM SPSS Statistics for Windows, Version 26.0. Armonk, NY: IBM Corp).

## Results

### Demographic profiles

Two hundred twenty two (222; 57.5%) of LT were due to IPF. When divided by their BMI at time of transplant, group 1 consisted of 13 (6%) patients, group 2 of 67 (30%) patients, group 3 of 79 (36%) patients and group 4 consisted of 63 (28%) patients (Table 1). Median BMI for g1 was 17 [interquartile range (IQR): 17, 18], for group 2 was 23 (IQR: 22, 24), for group 3 was 29 (28, 29.5) and for group 4 was 32 (31, 33). Patients in group 1 were significantly younger than patients in other groups (*p* < 0.01). Males comprised the majority of the patients in group 2, group 3 and group 4 (*p* = 0.01). Single LT comprised the majority of operation type in group 2, group 3 and group 4 and it was significantly higher than group 1 (*p* < 0.01; Table 1).

**Table 1:** Patient characteristics.

	All patients	Group 1	Group 2	Group 3	Group 3	*p*-value
BMI group		≤18.5	18.6–25	25–29.9	≥30	
Number of patients	222	13	67	79	63	
BMI (median; IQR)	27 (24, 30)	17 (17, 18)	23 (22, 24)	29 (28, 29.5)	32 (31, 33)	
Age (years)	56 ± 12	33 ± 9	54 ± 14	59 ± 9	56 ± 12	<0.01*
Gender (males)	159 (72%)	6 (46%)	39 (58%)	63 (80%)	51 (81%)	0.001*
Operation type (single LT)	159 (72%)	1 (8%)	39 (58%)	66 (84%)	53 (84%)	<0.01*
Lung allocation score (LAS)	40 (36, 47)	40 (35, 50)	38 (35, 45)	40 (36, 45)	39 (30, 50)	0.057
PA systolic pressure (mm Hg)	41 ± 12	33 ± 8	41 ± 9	41 ± 14	41 ± 13	0.49
CPB use	87 (39%)	8 (62%)	24 (36%)	27 (34%)	28 (44%)	0.20
CPB time (minutes)	175 ± 59	135 ± 26	172 ± 98	201 ± 69	353 ± 56	0.19
Prolonged ventilation^¥^	13 (6%)	2 (15%)	4 (6%)	3 (4%)	4 (6%)	0.63
ECMO**	4 (2%)	0	1 (1.5%)	1 (1.3%)	2 (3%)	0.26
Pneumonia	13 (6%)	0	5 (7%)	4 (5%)	4 (6%)	0.74
Primary graft dysfunction	5 (2%)	0	2 (3%)	1 (1%)	2 (3%)	0.85
Air leaks	87 (39%)	10 (77%)	27 (40%)	23 (29%)	27 (43%)	0.009*
Hospital length of stay (days)	14 (11, 18)	15 (14, 23)	14 (10, 19)	13 (11, 19)	14 (11, 21.5)	0.25
30-day readmissions	55 (25%)	2 (15%)	19 (28%)	21 (27%)	13 (21%)	0.64
Mortality						0.24^†^
30-day	10 (4.5%)	0	2 (3%)	3 (4%)	5 (8%)	
90-day	18 (8%)	0	4 (6%)	7 (8%)	7 (11%)	
1-year	37 (17%)	1 (8%)	6 (9%)	15 (19%)	15 (24%)	
5-year	79 (36%)	5 (38%)	17 (25%)	33 (42%)	24 (38%)	

BMI, body mass index; IQR, interquartile range; LT, lung transplant.

* Values are considered significantly different at *p* ≤ 0.05.

** All ECMOs were performed during lung transplant.

^¥^ Ventilation lasting >24 h post transplant.

^†^ Log rank test value.

### Clinical outcomes and post-operative survival

Patients were monitored for 5 years or until death. Median follow up time was 39 months (IQR: 13–76). There was no difference in prolonged ventilation, pneumonia and primary graft dysfunction between the groups (all *p *> 0.05). Significantly more air leaks occurred post-operatively in group 1 compared to other groups (*p *= 0.009). There was no statistically significant difference in hospital length of stay and 30-day readmissions between the four groups (*p* > 0.05; Table 1).

**Figure 1:**
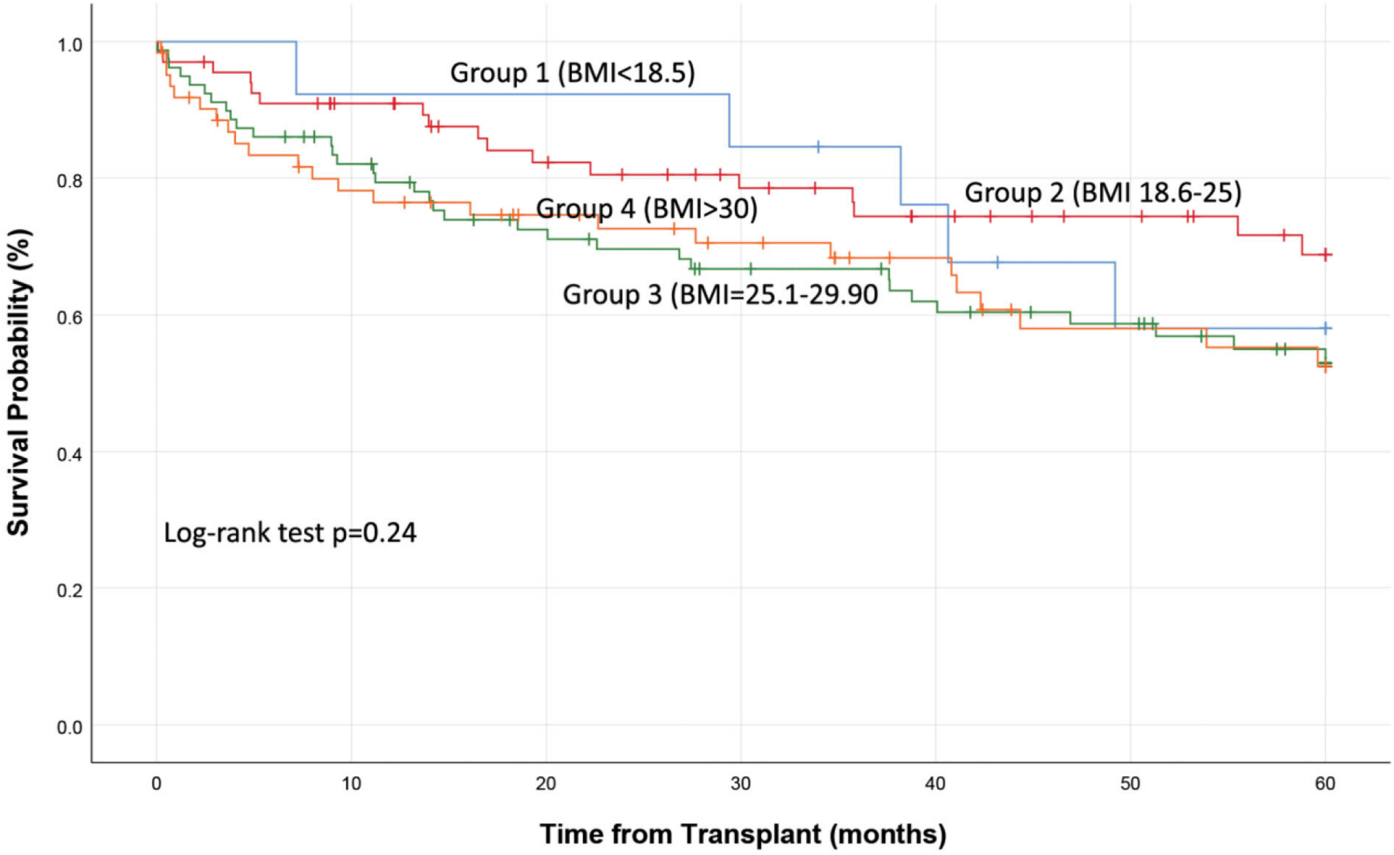
Kaplan Meier survival curve.

A total of 79 (35.5%) patients died by the end of study follow-up. Overall, 5 (6%) deaths occurred in group 1, 17 (21.5%) in group 2, 33 (42%) in group 3, and 24 (30%) in group 4. Mortality at 30- and 90-day, 1- and 5-year was similar between the groups (Log Rank test *p *= 0.24; Figure 1; Table 1). Cox-regression analysis was used to assess other possible risk factors that could influence the effect of BMI on mortality, including transplant type (single, double), lung allocation score, and age, diabetes and creatinine levels at surgery. None of these factors were shown to affect patient mortality (*p* > 0.05). Reasons for death included graft failure (24%), infection (23%), respiratory failure (14%), and malignancy (13%). Table 2 summarizes the breakdown of reasons for death for all four groups.

**Table 2:** Death etiology at 5 years post-transplant.

	Total	Group 1	Group 2	Group 3	Group 4
Total deaths	79	5	17	33	24
Cause of death					
Graft failure	19 (24%)	2 (40%)	5 (24%)	6 (18%)	7 (29%)
Primary failure	8	0	2	2	4
Chronic rejection	11	2	3	4	3
Infection	18 (23%)	2 (40%)	5 (6%)	7 (24%)	3 (13%)
Septicemia	8	1	1	4	1
Pneumonia	3	1	0	1	1
CMV	3	0	2	1	0
Fungal	4	0	2	1	1
Respiratory failure	11 (14%)	0	1 (6%)	5 (15%)	5 (21%)
Malignancy	10 (13%)	0	3 (18%)	4 (12%)	3 (13%)
Unknown	14 (18%)	0	1 (6%)	8 (24%)	5 (21%)
Cerebrovascular	1 (1%)	0	0	0	1 (4%)
Cardiovascular	1 (1%)	0	0	1 (3%)	0
Other	5 (6%)	1 (20%)	2 (12%)	2 (6%)	0

CMV, cytomegalovirus; Other, embolism, multi-organ failure, hypovolemic shock.

**Figure 2:**
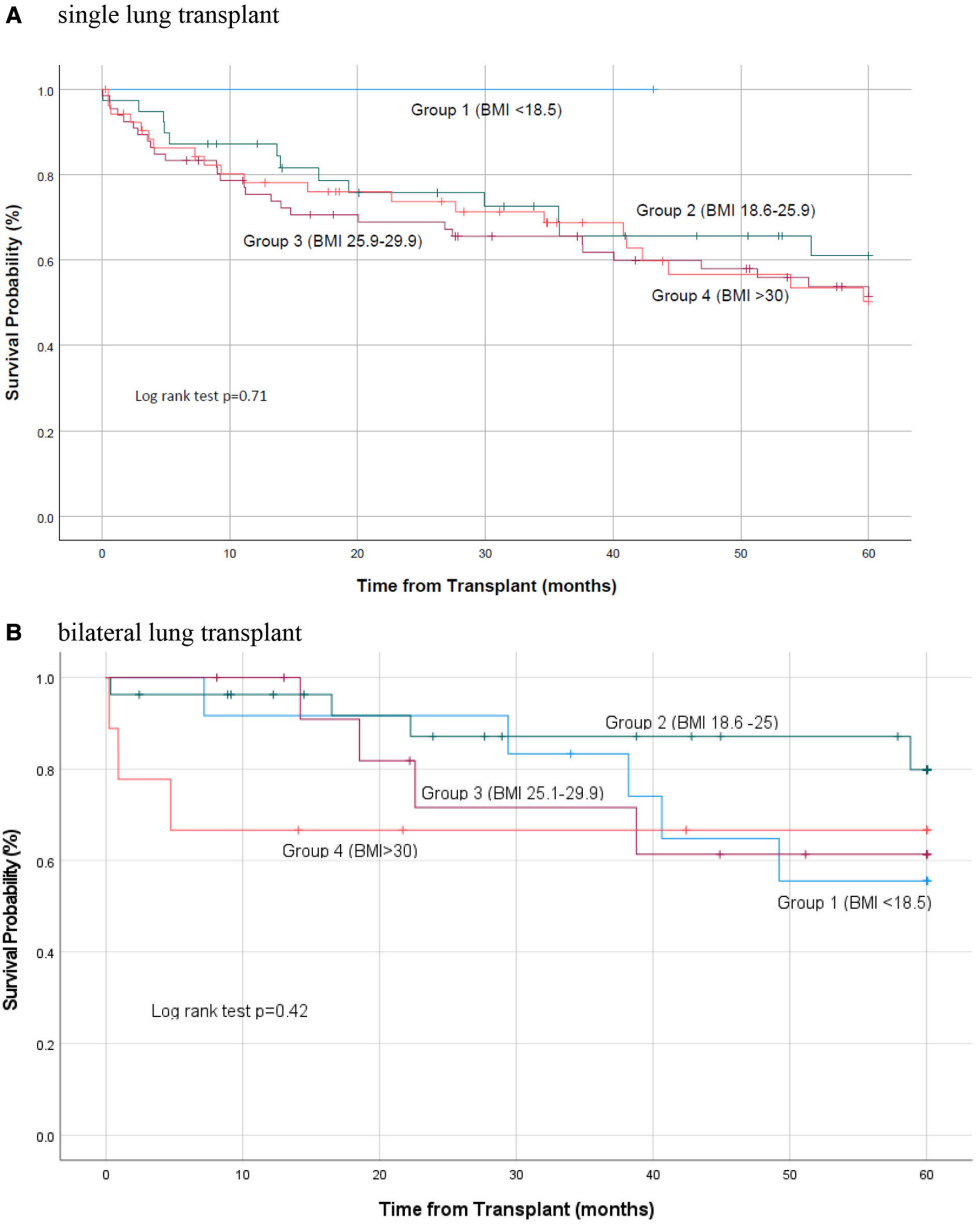
Kaplan Meier survival curve based on transplant type. (A) Single lung transplant; (B) Bilateral lung transplant.

Of the 79 total deaths, 37 of them occurred within 1 year post transplant. When divided by BMI groups 1 death occurred in group 1, 6 in group 2, 15 in group 3, and 15 in group 4 (Log Rank test *p *= 0.086). Reasons for death in the first year post-transplant included, graft failure (*n* = 8; 22%), infections (17%) and respiratory failure (25%). Overall, a majority of the deaths occurred in patients undergoing single lung transplant. Thirty-two of 36 (89%) deaths that occurred the first year were in patients receiving single LT (Table 3) We did not find a a statistically significant difference in mortality between different BMI groups in single or bilateral lung transplant patients (Table 3; Figure 2).

**Table 3:** Patient mortality based on transplant type.

	Single transplant					Bilateral transplant				
	Group 1	Group 2	Group 3	Group 4		Group 1	Group 2	Group 3	Group 4	
Patients	1	39	66	53	*p*-value	12	27	13	9	*p*-value
Mortality					0.71^†^					0.42^†^
30-day	0	1 (3%)	3 (5%)	3 (6%)		0	1 (4%)	0	2 (22%)	
90-day	0	3 (8%)	7 (11%)	5 (9%)		0	1 (4%)	0	2 (22%)	
1-year	0	5 (13%)	15 (23%)	12 (23%)		1 (8%)	1 (4%)	0	3 (33%)	
5-year	0	15 (38%)	29 (40%)	21 (40%)		5 (42%)	4 (15%)	4 (31%)	3 (33%)	

^†^ Log rank test value.

We also investigated whether there was a relationship between BMI and survival when BMI was considered a continuous variable. We found that BMI did not affect patient survival at 1- and 5-year post-transplant, even when considered as a continuous variable (*p* = 0.36; HR: 0.931, CI [0.79–1.08] and *p* = 0.59; HR: 0.97, CI [0.89–1.06] at 1-year and 5-year post-transplant respectively.

Median survival time of patients undergoing single LT was 37 (IQR: 11, 73) months and median survival time of patients undergoing double LT was 43 (IQR: 18, 82) months (*p* = 0.52). Forty percent (*n* = 63) of the patients undergoing single LT died during the study follow-up and 25% (*n* = 16) patients undergoing double LT died during the study follow-up (*p *= 0.037). There was not a significant relationship between BMI, operation type (single vs. double LT) and survival at 5-year post transplant (*p* = 0.08; HR: 0.30, CI [0.29–1.01].

## Discussion

The question whether BMI affects surgical outcomes and patient survival after LT has been the focus of several investigations. Although ISHLT guidelines recommend that BMI of 30 kg/m^2^ or greater, be considered a relative contraindication to lung transplantation, in practice, BMI thresholds for candidacy vary across centers. Conflicting results in the literature may drive these decisions. A large study using UNOS data found that both pre-transplant underweight and obesity contributed to up to 12% of deaths in the first year after transplantation and concluded that BMI is an independent risk factor for death after LT (19). Looking at a similar population another group found that overweight and class I obesity were not associated with early mortality (13). A study using the Scientific Registry of Transplant Recipients (SRTR) database, focused on individual BMI units and found that BMI is an independent predictor of mortality in lung transplant recipients at both 90 days and 1 year post-transplantation (12). They concluded that the patterns of BMI effect on patient survival do not fit into the predefined BMI categories.

In this study, we evaluated the impact of BMI on a select group of patients undergoing LT, patients with IPF. We used two approaches in our analysis. In the first approach, our study population was divided in groups based on their BMI. The relationship between BMI and both short- and long-term patient survival was not statistically meaningful. To avoid the bias of BMI grouping the second approach looked at BMI as a continuous variable. This approach resulted in similar findings that BMI did not affect short- and long-term patient survival in the select group of recipients with ILD.

A few studies have investigated BMI and survival in IPF patients. Alakhras and colleagues, examined 197 patients with a BMI <25, BMI 25–30 and BMI >30 (20). They found that there was an association between survival and BMI, and that increased BMI was associated with better survival. Of note, these patients did not undergo lung transplantation. Another study found that obese patients who receive bilateral LT might be at higher risk of 90-day mortality compared with patients of normal weight (21). Bilateral lung transplantation is the preferred option compared to single lung transplantation. Single lung transplantation is associated with poorer long-term outcomes (22). If we look at the latest SRTR annual report, even in 2010 bilateral lung transplant rates were significantly higher than single lung transplantation in USA (unlike the study cohort). If BMI really influences outcomes, the higher mortality rates seen in this cohort of patients may have confounded BMI effect on outcomes.

Our study has a few limitations, including its retrospective nature and the small sample size from a single institution. In addition, this observational study did not account for all other elements related to BMI that we could not capture such as frailty, change in weight over time, muscle mass, distribution of adipose tissue, targeted pre-transplant treatment, weight loss after surgery, rehabilitation characteristics for each group, etc. However, despite these limitations, this is the first study of ILD patients to analyze the effect of BMI on survival where BMI was also considered a continuous variable. This approach removes the bias on designation of BMI groups being somewhat arbitrary and may or may not reflect the actual effect on survival. The fact that both approaches gave similar results is reassuring.

Further exploration with a larger patient population may lead to additional insights into the association of BMI and patient survival and may help with management of patients afflicted with this disease.

## Data Availability

The raw data supporting the conclusions of this article will be made available by the authors, without undue reservation.
